# Supplementary material for: Durability of Humoral and Cellular Immunity after an Extended Primary Series with Heterologous Inactivated SARS-CoV-2 Prime-Boost and ChAdOx1 nCoV-19 in Dialysis Patients (ICON3)
Source: Vaccines (Basel). 2022 Jul 1;10(7):1064. doi: 10.3390/vaccines10071064 (PMC9323398; doi:10.3390/vaccines10071064)
Supplement: Supplementary file 1 [file vaccines-10-01064-s001.zip › vaccines-1736143-supplementary.pdf]

Supplementary Table S1. Changes in HMI and CMI responses in patients treated with HD or PD and in control participants after completing COVID-19 vaccination with the extended primary series, composed of a heterologous inactivated SARS-CoV-2 prime-boost and ChAdOx1 nCoV-19 vaccine as the third dose, beginning from baseline (M0) and continuing through 3 months (M3) and 6 months (M6) after vaccination.

Variable<br>N (%) or mean (SD)	HD<br>(n = 29)	PD<br>(n = 28)	Control<br>(n = 14)
Percent change in anti-RBD IgG titer from M0 to M3 [%], mean (SD)	−59.5%***<br>(27.9%)	−69.1%<br>(17.1%)	−80.2%<br>(11%)
Percent change in anti-RBD IgG titer from M0 to M6 [%], mean (SD)	−76.7%<br>(18.7%)	−84.1%<br>(9.6%)	N/A
Percent change in sVNT from M0 to M3 [%], median (IQR)	−6%<br>(−15% to −1%)	−5%<br>(−20% to −2%)	−6%<br>(−16% to −2%)
Percent change in sVNT from M0 to M6 [%], median (IQR)	−8%<br>(−15% to −1%)	−13%<br>(−30% to −5%)	N/A

Percent change in S1-specific T-cell response from M0 to M6 [%], median (IQR)	6% (−77% to 329%)	14% (−43% to 170%)	N/A

*** $p < 0.05$ (compared with control participants)

CMI, cell-mediated immunity; *HD*, hemodialyzed patients; *HMI*, humoral-mediated immunity; *IgG*, immunoglobulin G; *IQR*, interquartile range; *N*, number; *N/A*, not applicable; *PD*, peritoneal dialyzed patients; *RBD*, receptor-binding domain; *SD*, standard deviation; *sVNT*, surrogate virus neutralization test.
